# Metabolic Context of the Competence-Induced Checkpoint for Cell Replication in *Streptococcus suis*

**DOI:** 10.1371/journal.pone.0153571

**Published:** 2016-05-05

**Authors:** Edoardo Zaccaria, Jerry M. Wells, Peter van Baarlen

**Affiliations:** Host-Microbe Interactomics, Animal Sciences, Wageningen University, Wageningen, The Netherlands; Centers for Disease Control & Prevention, UNITED STATES

## Abstract

Natural genetic transformation is a transient, rapidly progressing energy-consuming process characterized by expression of the transformasome and competence-associated regulatory genes. This transient state is tightly controlled to avoid potentially adverse effects of genetic recombination on genome integrity during cell division. We investigated the global response of *Streptococcus suis* to exposure to the SigX competence-inducing peptide (XIP), and thus to the activation of the competence machinery, using time series analysis together with PCA analysis, gene clustering followed by heatmap visualisation, and GO enrichment analysis. We explored the possible regulatory link between metabolism and competence, and predicted the physiological adaptation of *S*. *suis* during competence induction, progression and exit using transcriptome analysis. We showed that competence development is associated with a suppression of basal metabolism, which may have consequences for the microbe's resilience to fluctuations in the environment, as competence is costly in terms of use of energy and protein translation. Furthermore our data suggest that several basal metabolic pathways are incompatible with activation of competence in *S*. *suis*. This study also showed that targeting specific pathways during the development of competence, might render *S*. *suis* more vulnerable toward novel antibiotic therapies.

## Introduction

The *Streptococcus suis* pheromone-induced competence regulon was characterised using a temporal transcriptomics approach, revealing that competence in *S*. *suis* is indeed transient as shown in previous studies [1] and characterised by tightly controlled expression of the transformasome and associated regulatory genes (Zaccaria et al., 2015 in publication). Natural genetic transformation is associated with internalization and chromosomal recombination of exogenous DNA with the genome, enabling bacteria to obtain new genetic traits that may improve fitness in changing environmental conditions, including those involving avoidance of host immune defences. Competence-inducing conditions in Gram-positive bacteria are generally related to stress challenges, circumstances, which in nature would select for adaptation and resilience and eventually, may improve fitness [2]. The *comCDE* operon of *S*. *pneumoniae* is located close to the chromosomal replication origin and as a consequence antibiotics that target DNA replication cause stalling of the replication fork, leading to increased copy number of *comCDE* and competence induction [3]. Under laboratory conditions, competence is influenced, among others, by temperature, growth medium, pH and the concentrations of magnesium and calcium [4]. Integrity of the plasma membrane or of the bacterial cell wall, perception of external stress, nitrogen concentration and cell density are some of the stimuli regulating, positively or negatively, the activation of competence [5,6]. Interestingly, pheromone induction of competence in proliferating *S*. *mutans* leads to the competence state in only a proportion of the bacteria, the remaining population undergoing cell death [7]. However, the interaction of these processes with ComCDE or ComRS and how they may participate in the modulation of the physiological state of the cells and with decisions to commit to competence or to cell death are not well understood.

Despite the evolutionary advantages of natural competence, uptake and incorporation of "foreign" DNA into the recipient genome is not without potential risks. Recombination of DNA with different genetic content may cause a gain or loss of functionalities. Additionally, DNA recombination during the process of elongation and chromosome replication is potentially dangerous to genome integrity. Furthermore, competence development may negatively affect the organism's basal metabolism and fitness as the process is costly in terms of use of energy and protein translation. The biological relevance of the possible physiological changes that might co-occur with competence development prompted us to investigate the possible regulatory link between metabolism and competence development, and to find out more about the physiological adaptation of bacteria upon induction of the competence state.

One molecular link between competence and metabolism is the small peptide pheromone SigX-inducing-peptide (XIP), that, in a number of streptococcal species, is transported into the intracellular environment by the general peptide transporter system designated Opp [8,9]. Opp transporters are located in the plasma membrane, and their main function is to take peptides from the extracellular environment to serve as sources of carbon and nitrogen that are necessary for bacterial proliferation [10], thus providing some contextual information on extracellular availability of nutrients. The use of a general peptide transporter for uptake of the pheromone may not be coincidental as it could be an indirect sensor of available oligopeptides and thus competitors occupying the same niche. For example, the addition of a high concentration of tryptone extract (but not casamino acids), to complex media can inhibit XIP induction of competence in *S*. *mutans* [11] and in *S*. *thermophilus* [12].

Temporal transcriptome data obtained during competence development (Zaccaria et al., 2015 in publication, see Methods) were analyzed under the hypothesis that it would reveal the metabolic context of bacteria entering, executing and exiting the competence state. We investigated which basal processes appeared to be induced during competence, and which processes were incompatible with bacterial proliferation. Such analyses would, in addition to unravelling the genetic regulation of competence in *S*. *suis*, hint at why competence induction can be potentially unfavourable for the bacteria. This is not only of fundamental interest but might also contribute to the development of future therapeutic applications, for instance induction of competence might render *S*. *suis* bacteria more vulnerable to antibiotics and the host immune response.

## Materials and Methods

### Bacterial strains and culture conditions

The virulent *S*. *suis* serotype 2 strain S10 was used in this study. *S*. *suis* S10 genome is 99% identical to the genome of *S*. *suis* 2 strain P1/7 [13], a sequenced reference strain of which the genome had been annotated previously [14]. *S*. *suis* was grown at 37°C at 5% atmospheric CO_2_ in Todd Hewitt Broth (THB, Thermo Scientific, Oxoid) with 1.2% of agar (BD) if solid medium. Growth phase was determined by measurement the optical density (OD_600nm_) using SpectraMax M5 reader (Molecular Devices LLC).

### RNA extraction

Induction of competence was performed as previously described [1]. Briefly *S*. *suis* S10 was grown until OD_600nm_ 0.04. Thirty-five ml of culture was collected and donor DNA (pNZ8048, 350 μg) in EB buffer (10 mM Tris-Cl, pH 8.5) was added to the bacteria along with synthetic XIP (GNWGTWVEE) at a final concentration of 250 μM. For the control same quantity of culture was collected and donor DNA added. Ten ml of the induced cultures were collected after 5, 15 and 45 minutes of XIP addition. Ten ml of the uninduced cultures were collected at 15 and 45 minutes. The samples were centrifuged for 2 min at 8000 g and the pellet resuspended in 2.5 mL PBS plus 5 mL RNAprotect buffer (Promega). After 5 minutes of incubation the bacteria were again collected by centrifugation and immediately frozen in liquid nitrogen until further use. The frozen pellet was dissolved in 110 μl of TE containing 1.25 μg/mL proteinase K and 15 ug/ml lysozyme and incubated for 10 minutes. Then 700 μL RLT buffer (Promega) containing 7 μl of freshly added β-mercaptoethanol was added to the dissolved pellet and the bacteria were lysed using a FastPrep-24 (MP Biomedicals, Solon, OH) for 6.0 m/sec at 20 sec. Total RNA was purified using RNeasy Mini Kit (Quiagen). The quality and the concentration of RNA were assessed with an Experion System (Bio-Rad) and by analysis of the A260/A280 ratio (NanoDrop 8000 UV-Vis Spectrophotometer). cDNA was synthesized with SuperScript III Reverse Transcriptase kit (Invitrogen) using Aminoallyl-dUTP as a source dUTPs and purified with Illustra CyScribe GFX Purification Kit (GE Healthcare). The cDNA was labelled with CyDye Post-Labeling Reactive Dye Pack (GE Healthcare).

### Microarray transcriptome analysis

An *S*. *suis* oligoarray (8×15 K) containing *in situ* synthesized 60-mers was produced by Agilent Technologies (Santa Clara, USA), based on the genome sequence of *S*. *suis* P1/7 [14]. A total of 7651 unique 60-mers having a theoretical melting temperature of approximately 81°C and representing 1960 ORFs were selected as described [15]. The majority (91%) of genes were represented by 4 probes with the remaining probe distribution as follows: 3 (4%), 2 (2%), or 1 probe (3%). Twenty-five putative genes were not represented on the array because no unique probe satisfying the selection criteria could be selected. Co-hybridization with labelled cDNA probes was performed on the oligonucleotide arrays at 42°C for 16 h in Slidehyb#1 (Ambion, Austin, USA). The data were normalized using Lowess normalization [16] as available in MicroPrep [17] and corrected for inter-slide differences on the basis of total signal intensity per slide using Postprep [17]. Significance of differential gene expression was based on false discovery rate (FDR) values lower than 0.05. The data discussed in this publication have been deposited in NCBI's Gene Expression Omnibus and are accessible through GEO Series accession number GSE74507.

### Transcriptome data mining

Short Time-series Expression Minor (STEM) [18] was used to cluster and compare gene expression intensities to identify genes of which the expression was significantly changed across all the time-points.

Cluster 3.0 (http://bonsai.hgc.jp/~mdehoon/software/cluster/software.htm) was used to filter the data for the most differentially expressed genes using the following parameters: standard deviation: 250; at least 4 observations with absolute value higher than 20; subtraction between maximum and minimum: 200.

The heatmaps were generated by MultiExperimental Viewer (MeV) program (http://www.tm4.org/mev.html) [19].

For all genes and proteins identified in the *S*. *suis* P1/7 genome, and KEGG pathway annotations were obtained using the BLAST2GO software (www.blast2go.org) [20] including annotations based on terms obtained from EBI using the InterPROScan feature [21] that is part of BLAST2GO while GO terms over-represented were calculated via the integrated FatiGO package [22].

## Results and Discussion

### Half the *S*. *suis* transcriptome is differentially expressed in response to competence induction

Based on our initial competence kinetics study [1], we examined the transcriptome of *S*. *suis* in response to competence induction after 5, 15 and 45 minutes exposure to XIP. The data discussed in this publication have been deposited in NCBI's Gene Expression Omnibus and are accessible through GEO Series accession number GSE74507. Before providing a detailed analysis of the transcriptome changes across the competence timeline, we will provide an overview of the transcriptome changes at the relevant timepoints.

After 5 minutes of exposure to XIP, *S*. *suis* cells become competent, thus produce the necessary machinery able to take up and recombine exogenous DNA [1]. After 5 minutes, almost half the *S*. *suis* transcriptome (918 of the 1969 known ORFs, 46.6%) showed a statistically significant differential expression (from now on, "differentially expressed" implies: statistically significant differential expression) compared to the control; 82% of the differentials were downregulated. At 15 minutes, when transformation efficiency reaches its peak, 333 (16.9%) genes were differentially expressed. At 45 min, when competence is lost, 115 genes (5.8%) were differentially expressed (Table 1). These data show that competence induction has a major impact on *S*. *suis* gene expression, with more than 80% of the differentials being suppressed after 5 min of competence induction. This differential regulation emphasises that competence has a major impact on *S*. *suis* regulatory processes, likely including processes involved in metabolism and physiology. This major reorientation of gene expression directly following competence induction in *S*. *suis* differs from transcriptomic analyses performed in other streptococcal species [23–25]. After competence induction in *S*. *gordonii* only 35 and 127 differentially expressed genes were identified at 5 and 15 minutes respectively [23]; similar results were obtained in *S*. *pneumoniae* [24,25]. *S*. *gordonii* and *S*. *pneumoniae* use a different proximal switch for induction of *comx* expression and might also differently regulate the competence-induced checkpoint for cell replication. Moreover, the growth phase of competence induction differs, being lag phase for *S*. *suis* but exponential phase for *S*. *gordonii* and *S*. *pneumoniae* [5,6]. The finding that more than 80% of the differentials were downregulated prompted us to identify suppressed cellular processes and evaluate what bearing these may have on bacterial function and persistence. We wanted to analyse the physiological impact that competence induction may have on bacterial metabolism. The global response of *S*. *suis* to exposure to XIP, and thus the activation of the competence machinery, was analysed using STEM time series analysis together with PCA-heatmap analysis and GO enrichment analysis. These analyses enabled us to identify sets of co-regulated genes of which the expression significantly changed during competence induction,—development and—termination and to identify the biological processes that were differentially regulated at the onset of, and exit from, competence. Transcriptome analysis by STEM is a useful way to achieve this since STEM analysis clusters genes based on similar gene expression values across all the time-points, comparing expression profiles to pre-modelled trends, and calculating GO enrichment for each cluster (see Methods) [18].

**Table 1:** Number of genes with expression significantly changed at 5, 15 and 45 minutes compared with the control.

	5 Min	15 Min	45 Min
**Up regulated**	163	162	78
**Down regulated**	755	171	37

### STEM analysis

In the next section, we outline the processes that were differentially regulated during competence, with special attention for regulation of bacterial metabolism. Because competence involves DNA repair, a process that is largely incompatible with whole-genome replication, we hypothesised that competence is incompatible with most cellular processes apart from pathways involved in DNA uptake, DNA processing, recombination and repair. Thus, we expected among others, to identify expression profiles of genes that were downregulated at the 5-min time-point, when competence was activated.

**Fig 1:**
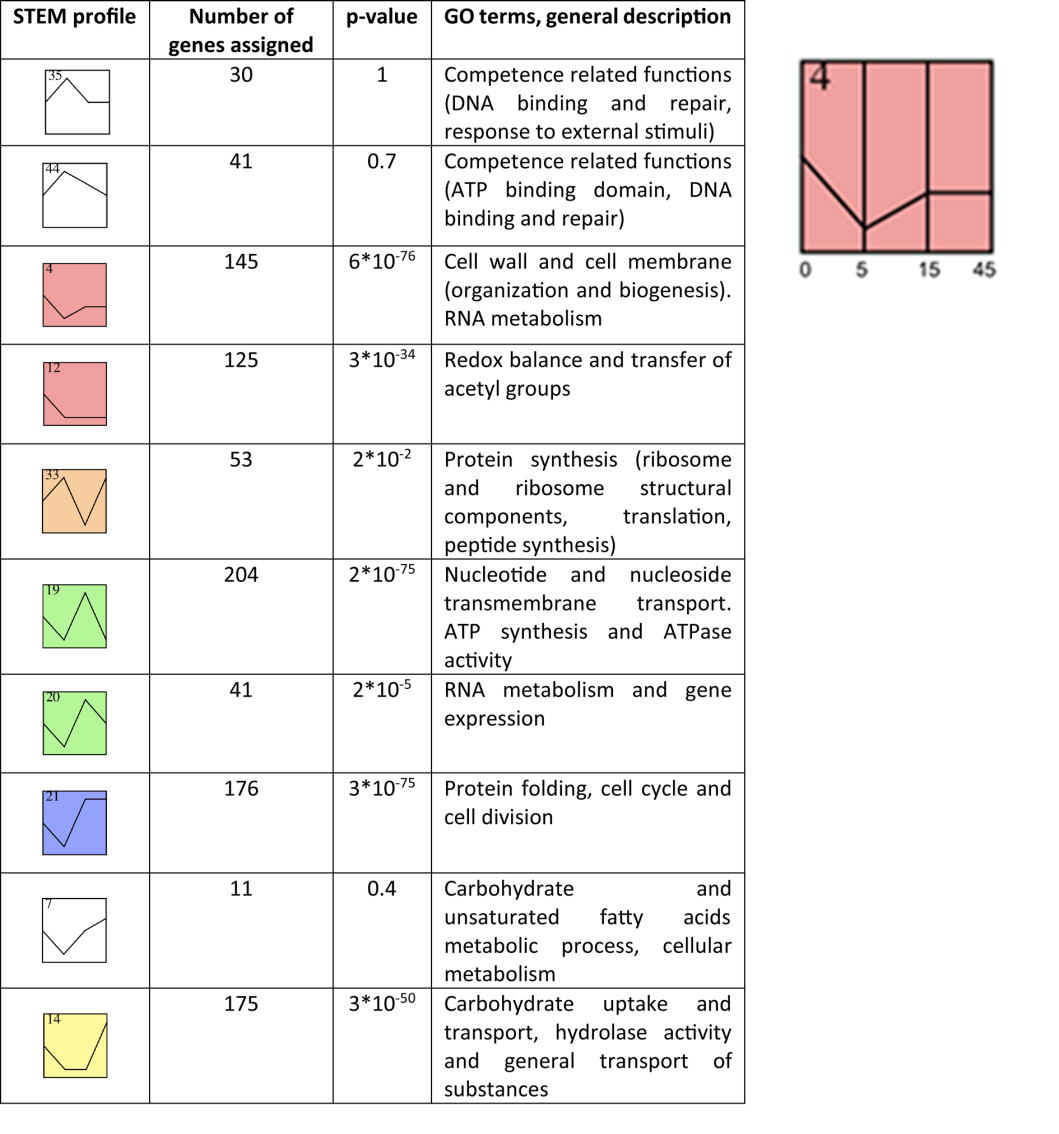
Overview of STEM profiles representing gene clusters differentially modulated throughout induction of competence in *S*. *suis*. Coloured profiles had a statistically significant number of genes assigned. Non-white profiles of the same colour represent profiles that could be grouped into a single cluster. The enlarged STEM profile shows the time-point locations; for all profiles, the time-points have been schematically displayed in this manner.

STEM analysis identified genes of which the expression had significantly changed at 5 min, when bacteria were entering competence state, at 15 minutes (peak competence) and 45 minutes (exit from competence), and retrieved their respective gene ontology (GO) (geneontology.org) terms. These GO terms helped us to identify the biological processes that had changed across the three time-points, from the early stage of competence at 5 min after induction to the shutdown of competence at 45 min. The possible link between competence and metabolism led us to hypothesise that in our transcriptome data, we should be able to find clusters of co-expressed genes with GO terms involved in metabolism, energy state and cellular fate. Moreover, we reasoned that consistently downregulated genes would be annotated with GO terms pointing at those cellular processes that were repressed during competence. We set out to investigate clusters of co-expressed genes, expected to together carry out specific tasks, in a hypothesis-driven manner, by postulating cellular functions that can be expected to be induced or repressed before, around and after the induction of natural competence. In the upcoming section, we list expression profiles representing significantly altered genes and their enriched GO terms resulting from STEM analysis (Fig 1).

We first searched for profiles including the known competence genes.

Profile 35 and 44 include the competence genes, including those under direct control of ComX. As predicted, the associated GO terms: "DNA binding", "response to external stimuli" and "DNA metabolic process" reflect common functions of natural genetic transformation. The associated GO term "ATP binding" also shows that energy metabolism is significantly altered during competence. White profiles do not contain a statistically significant number of genes showing that trend [18]. Note that absence of statistical significance does not imply that the trend bears no biological relevance.

Profile 4 contained genes that were strongest downregulated during 5 min of competence. The genes belonging to this profile participated in cell wall, cell division and RNA metabolism, suggesting that gene expression and bacterial proliferation were suppressed when competence was induced. Interestingly genes associated with the GO term "oxidoreductase activity", representing the redox processes that are at the basis of biochemical reactions, were included in this cluster. Note that this should be interpreted as a significantly lower expression compared to the non-competent state, rather than no gene expression during competence induction at all.

Profile 12 represents a second cluster of co-expressed genes with significantly reduced expression throughout the competence period. Note that 4 and 12 display the same colour; this indicates that these profiles include a statistically significant number of genes that could be grouped into a single larger cluster characterised by an expression profile encompassing the two separate profiles. The genes included in profile 12 were involved in redox balance and transfer of acetyl groups, a common event in biological metabolic synthetic—and catabolic reactions. Together the profiles 4 and 12 encompass 270 significantly lower expressed genes that represent more than one third of the 80% of significantly differentially downregulated genes at 5 minutes with strongly overrepresented GO terms, compared with the uninduced samples. The expression profiles of the genes included in profiles 4 and 12 support the notion that the competence state is characterised by a suppression of general metabolism.

Profile 33 included genes that were upregulated at 5 and 45 minutes and downregulated at 15 min, at the peak of transformability. The genes showing this expression pattern were encoding structural components of the ribosome and involved in rRNA binding, gene expression and protein translation. Suppression of ribosome gene expression is a straightforward way to suppress translation and *de novo* cellular protein production, thereby suppressing the activation of any pathway that relies on *de novo* protein production.

We were interested which genes were necessary for competence to proceed; we reasoned that at the peak of competence induction, defined as the highest number of colonies after competence-induced transformation, a specific set of genes would be induced. Profile 19 was of interest to us as it included genes that were downregulated at all time-points except at 15 min. This profile included genes involved in transmembrane transport and (purine) nucleoside and nucleotide transport and six transcriptional regulators of which SSU0388 encodes an orthologue of a protein induced by cell wall stress conditions and SSU1826 encodes an orthologue of a protein involved in competence in other streptococci [26].

We also observed a cluster of genes that were induced at peak of competence induction but that were not downregulated at 45 min as in profile 19, but of which the expression was restored to the uninduced state. This cluster included genes participating in RNA metabolism and gene expression. This profile, together with the previously reported profiles, suggests that expression of at least some genes is induced between peak and shutdown of competence. This profile includes a PadR-like transcriptional regulator, a protein that is usually part of the response to phenolic acids, facilitating the bacteria to adapt to stressful environments [27,28]. We searched for other clusters of genes induced at peak competence.

Profile 21 included genes that were suppressed at 5 minutes, induced between 5 and 15 min and remained induced between 15 and 45 min. The genes in this profile participated in protein folding and the cell cycle. This cluster together with the previous two clusters suggested that at 15 and 45 minutes, genes involved in specific metabolic pathways including protein folding and involved in the cell cycle had been induced. This was of interest since this hinted at induction of cell proliferation at the later time-points, whereas these processes had been suppressed at the onset of competence. Taken together, the trends and GO term enrichment reported by profiles 4, 12 and 33 (earlier timepoint upon competence induction) and by profiles 19, 20 and 21 (halfway and late timepoint upon competence induction) suggest that a large subset of basal metabolic genes had been downregulated at the onset of competence and that a second subset had been induced between the peak and shutdown of competence.

Next, we searched for a gene profile that included genes downregulated at the earlier time-points and upregulated at later time-points. The small cluster 7 contained genes continuously induced after 5 min that were involved in metabolism of carbohydrates and unsaturated fatty acids, processes that play roles in basal metabolism and bacterial proliferation, more specifically, during the synthesis of novel membranes.

Profile 14 contained genes that had been downregulated until 15 min (peak of competence) but were strongly induced between 15 and 45 minutes. These genes were involved in carbohydrate uptake and transport across the membrane, and amide metabolic processes that commonly involve the modification of carboxylic acids including amino acids and organic acids via biochemical reactions that include formation of amides. Profile 14, together with the previous two profiles represent clusters of genes with distinct metabolic functions.

The STEM analysis results suggested that competence of *S*. *suis* is characterised by two main stages. A first stage includes the timepoints 5 and 15 minutes when competence is induced and the competence state develops (peaks) that is characterised by a general repression of gene expression, protein production, cell cycle and metabolism (note that 80% of the differentials had been downregulated from the onset of competence). A second stage includes the timepoints 15 and 45 minutes upon exit from competence, characterised by an induction of genes involved in protein folding, cell cycle and fatty acid metabolism. It is not possible to precisely identify when each specific biochemical process started. To explore this further, we filtered the transcriptomes for those genes that showed the highest differences in expression between the three time-points, using the raw gene expression values, performed principal component analysis (PCA) and plotted the PCA output on a heatmap representing raw gene expression of the filtered genes.

### PCA and cluster analysis shows that initiation, peak and exit of competence are regulated by distinct gene clusters

**Fig 2:**
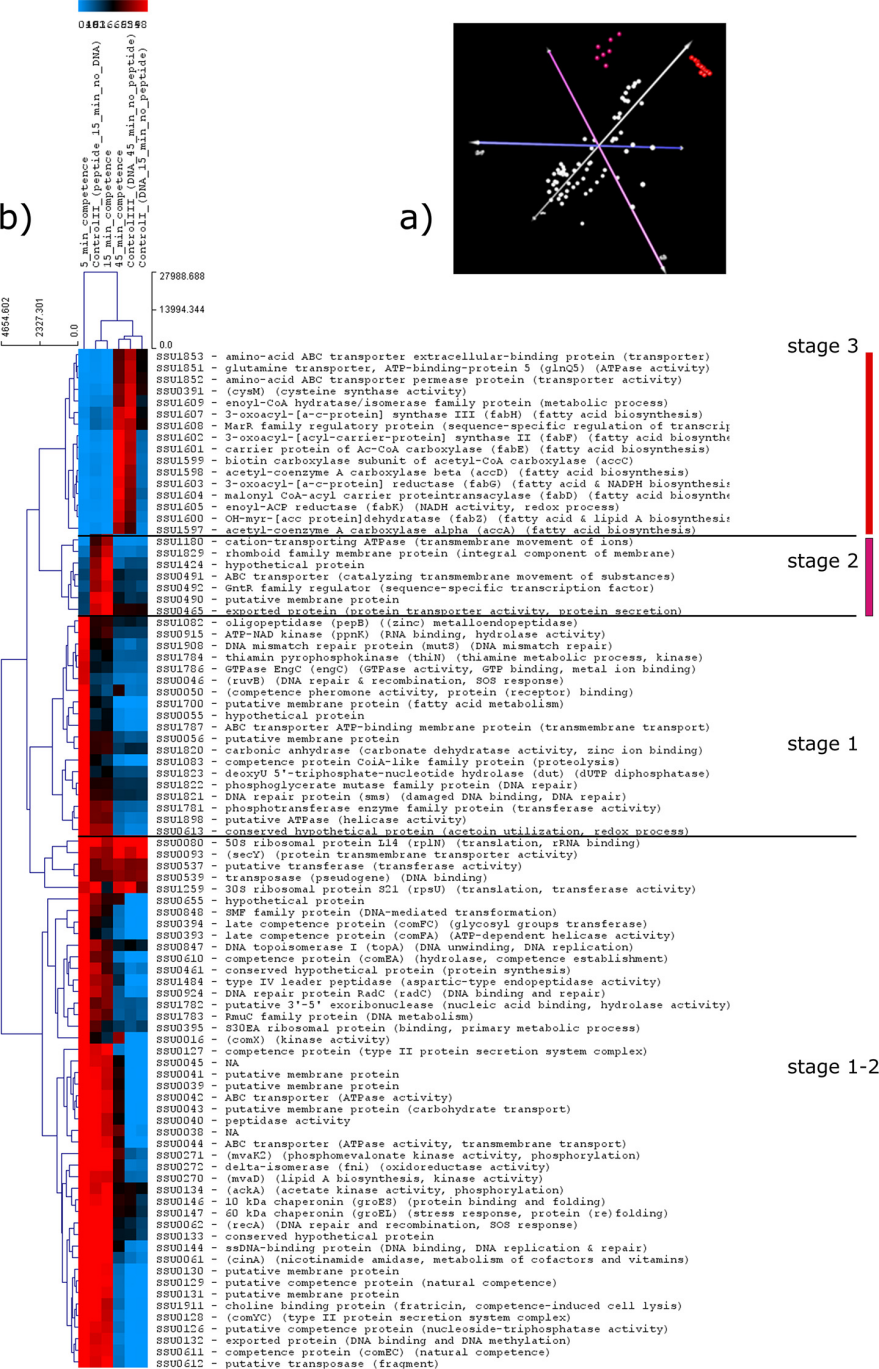
Most differentially expressed genes upon competence induction in *S*. *suis*. a) Principal Component Analysis (PCA) plot showing three major clusters (in white, red and purple). Red and purple clusters correspond with distinct clusters in the heatmap indicated by the same colours. b) Heatmap displaying the 88 most differentially expressed genes clustered using average linkage and Euclidian distance into 3 distinct time-dependent stages.

STEM analysis, in particular profile 14, 7, 4 and 12, suggested that competence induction might be incompatible with bacterial growth at 5 minutes but not, or less so, at later time-points. To investigate this notion, we expected to be able to find clusters of genes involved in cell proliferation strongly induced at especially 45 minutes. PCA analysis successfully separated the dataset into six components, with the first 2 components explaining 90,9% of the total variation and the first 3 components explaining 97,8% (Fig 2A). 2D and 3D analysis of the PCA plot showed that at least three major clusters could be identified, coloured white, red and purple in Fig 2A. We generated heatmaps to display gene expression as normalised values, not as ratio data, to identify genes that were highly expressed. Pre-filtering genes (see Methods) resulted in a set of 88 genes, 4.5% of the *S*. *suis* genome, including genes encoding proteins with diverse metabolic functions, transcriptional regulators, heat-shock proteins and chaperones, proteases, and genes involved in competence including the gene SSU0050 encoding the competence peptide pheromone (Fig 2B). We inferred that the presence of these key competence (and competence-related) genes in the 88-gene set indicated that the filtering of genes had resulted in a set that was still biologically relevant to competence. The 88 genes were clustered using average linkage and Euclidian distance (see Methods) and displayed in a heatmap. Visual inspection of gene expression across the heatmap showed that these 88 genes had clustered into distinct time-dependent stages, 1–3 (Fig 2B). Plotting the three major PCA clusters onto the heatmap showed that the red and purple clusters corresponded with distinct clusters in the heatmap (Fig 2B). The combined PCA and heatmap clusters showed that the initiation and peak competence gene clusters contain the white and purple PCA clusters and span stages 1, 1–2 and 2, and that the red PCA cluster corresponds to a separate heatmap cluster, stage 3. Stage 3 includes the fatty acid metabolism operon, and all genes in this cluster were only strongly expressed upon competence exit. This PCA and heatmap analysis of the most differentially expressed genes corroborates the notion that the earlier competence time-points are characterised by gene expression profiles that are very different from the later time-point, when the competence state is shut down. The PCA and heatmap analysis also shows that genes involved in cell proliferation, the fatty acid metabolism genes of the red PCA cluster and the stage 3 cluster of the heatmap, are only strongly expressed when the bacteria have exited the competence state [1]. The STEM and PCA analyses therefore support the hypothesis that induction and execution of competence is incompatible with bacterial proliferation. We decided to investigate this prediction more extensively using a GO term enrichment approach.

### Gene enrichment analyses corroborate the profound differences between earlier and later stages of competence

To further examine our hypothesis that competence activation induces a cell proliferation blockade, we analysed which GO terms were over-represented for each time-point using Blast2GO and its integrated FatiGO package [20,29] (see Methods) (Table 2). For each time-point, we compared significantly up- or downregulated genes and calculated which GO terms, associated with the up- or downregulated gene sets, were significantly enriched or over-represented. For convenience, we report a selection of the GO terms that were statistically over-represented. At 5 and 15 minutes, the down-regulated genes carried GO terms associated with cell proliferation and basal metabolism whereas GO terms associated with DNA binding were over-represented in the up-regulated gene sets. Interestingly, genes carrying the GO term "ATP binding" were up-regulated, highlighting that the competence state is an energy-demanding process, probably linked with transformasome formation, active DNA uptake, processing, recombination and repair. The higher demand for ATP binding reflect the high energetic costs that are commonly ascribed to transformasome formation and DNA uptake and recombination. We hypothesise that, in the competence state, energy-yielding molecules are partially depleted and responses to environmental changes will be slower, which may be relevant for future *S*. *suis* control strategies (see Concluding Remarks below).

**Table 2:** Selection of over-represented GO terms for each time-point.

	Downregulated		Upregulated	
Timepoint	GO term	P-value	GO term	P-value
**5 minutes**	Cellular catabolic process	77.56E^-06^	Structure-specific DNA binding	2.35E^-04^
	Lipid biosynthetic process	88.31E^-05^	DNA binding	2.49E^-04^
	Cellular biosynthetic process	11.13E^-04^	ATP binding	7.65E^-04^
	Cell wall organization or biogenesis	11.33E^-04^	Hormone biosynthetic process	4.00E^-03^
	Regulation of primary metabolic process	66.47E^-04^		
**15 minutes**	Fatty acid biosynthetic process	11.78E^-10^	ATP binding	3.53E^-04^
	Carboxylic acid biosynthetic process	77.29E^-09^	Hormone biosynthetic process	3.55E^-04^
	Organic acid metabolic process	11.40E^-06^	Carbohydrate derivative binding	6.37E^-04^
	Single-organism biosynthetic process	22.41E^-05^	DNA binding	2.75E^-03^
	Acetyl-CoA carboxylase complex	55.74E^-05^	DNA mediated transformation	5.37E^-03^
**45 minutes**	Membrane	11.84E^-02^	Fatty acid biosynthetic process	5.81E^-16^
	Regulation of transcription, DNA-templated	22.67E^-02^	Monocarboxylic acid biosynthetic process	1.62E^-15^
	Regulation of RNA metabolic process	44.73E^-02^	Lipid biosynthetic process	5.85E^-14^
			Ligase activity, forming carbon-carbon bonds	1.38E^-06^
			Single-organism biosynthetic process	7.01E^-03^

At 45 minutes, the over-represented GO-terms were associated with activation of metabolic processes, cell growth and proliferation; these processes had been suppressed at the earlier time-points. The low number of genes down-regulated at 45 minutes: 37, representing 1.9% of the *S*. *suis* genome, exemplifies how similar bacterial gene expression was at 45 minutes compared to pre-competence induction conditions. In contrast, 38% of the transcriptome had been downregulated at the 5-min time-point. The STEM, PCA and GO enrichment results are in agreement and show that the competence state of *S*. *suis* is characterised by an initial suppression of processes associated with bacterial proliferation and activation of the natural transformation machinery, followed by induction of fatty acid metabolism, protein translation and cell division at 45 minutes, effectively re-establishing bacterial growth. These results show the massive impact competence activation has on *S*. *suis* physiology and metabolism. At 5 and 15 min after competence induction ribosome function and basal metabolism were suppressed, compatible with a delay or checkpoint in DNA replication and cell division. Thus in *S*. *suis*, competence induction reduces cell proliferation and bacterial growth despite the bacteria growing in a very rich medium and at a bacterial growth phase usually characterized by fast replicating bacteria. The delay or checkpoint in DNA replication and cell division during competence is expected since homologous recombination of the transforming DNA during chromosomal replication may lead to replication errors [30,31]. In *S*. *pneumoniae*, activation of the chromosome segregation machinery negatively influences competence development [32] showing that also in species in which the competence activation machinery differs from the machinery described for *S*. *suis* [1], cell growth and competence act antagonistically. In *B*. *subtilis* activation of ComCG prevents cell elongation and cell division before exit from competence [33]. A homologue of ComGC, ComYA, is induced by the competence pheromone in *S*. *suis*; it is not known if ComYA is required for checkpoint control of cell division.

At 45 minutes after competence induction, *S*. *suis* gene expression features among others activation of the fatty acid and carbohydrates metabolism, enabling cell proliferation and bacterial growth, compatible with the physiology that is typical for bacteria growing *in vitro* under good growth conditions.

## Concluding Remarks

Our data highlight that in *S*. *suis*, as in other bacteria with a functioning competence mechanism, basal metabolic pathways are incompatible with activation of competence. The 5-min competence time-point is characterised among others by cellular activities involving ATP binding and energy-costly processes such as transformasome formation, depleting the bacteria's energy pool. Thus we can hypothesise that induction of competence and energy depletion might render bacteria more vulnerable to antimicrobial therapy and the host immune defences. Moreover, our study proposes that upon competence activation, *S*. *suis* is characterised by an unfavourable energy balance and not well suited to control its redox balance since key antioxidant genes as thioredoxin and superoxide dismutase are hardly expressed during the competence state. Thus, it is possible that competent bacteria are more susceptible to reactive oxygen species which are produced by neutrophils and macrophages following bacterial contact. We consider that it might be of interest to investigate if induction of competence of *S*. *suis* could render the bacteria less virulent during infection. Indeed, Oggioni and collaborators’ studies [34,35] provide an indication that this may be a realistic scenario. They evaluated the effect of competence induction on virulence in a mouse model of pneumococcal sepsis and demonstrated a significant increase in mouse survival and a reduction in blood *S*. *pneumoniae* counts. However, induction of the competence system increased virulence if the bacteria were in a biofilm-like state, e.g. as described in pneumonia. Our transcriptome analyses have provided us with pathways that may be more vulnerable to small molecule inhibitors when targeted during the competence state, and that interfering with these pathways may contribute to suppress proliferation of *S*. *suis*. Blocking essential pathways, rather than blocking individual genes, might decrease the incidence of resistance, since chances of developing resistance in all the pathway genes, at the same time keeping the pathway functional, are low. Our transcriptome data may thus contribute to developing novel compounds that control excessive outgrowth of *S*. *suis* in pigs.

## References

[1] Zaccaria E, van Baarlen P, de Greeff A, Morrison DA, Smith H, et al (2014) Control of competence for DNA transformation in streptococcus suis by genetically transferable pherotypes. PLoS One 9: e99394 10.1371/journal.pone.0099394 24968201 PMC4072589

[2] LaSarre B, Federle MJ (2013) Exploiting quorum sensing to confuse bacterial pathogens. Microbiol Mol Biol Rev 77: 73–111. 10.1128/MMBR.00046-12 23471618 PMC3591984

[3] Slager J, Kjos M, Attaiech L, Veening JW (2014) Antibiotic-induced replication stress triggers bacterial competence by increasing gene dosage near the origin. Cell 157: 395–406. 10.1016/j.cell.2014.01.068 24725406

[4] Morrison DA (1997) Streptococcal competence for genetic transformation: regulation by peptide pheromones. Microb Drug Resist 3: 27–37. 9109094 10.1089/mdr.1997.3.27

[5] Fontaine L, Wahl A, Flechard M, Mignolet J, Hols P (2015) Regulation of competence for natural transformation in streptococci. Infect Genet Evol 33: 343–360. 10.1016/j.meegid.2014.09.010 25236918

[6] Johnston C, Martin B, Fichant G, Polard P, Claverys JP (2014) Bacterial transformation: distribution, shared mechanisms and divergent control. Nat Rev Microbiol 12: 181–196. 10.1038/nrmicro3199 24509783

[7] Wenderska IB, Lukenda N, Cordova M, Magarvey N, Cvitkovitch DG, et al (2012) A novel function for the competence inducing peptide, XIP, as a cell death effector of Streptococcus mutans. FEMS Microbiol Lett 336: 104–112. 10.1111/j.1574-6968.2012.02660.x 22900705 PMC4669055

[8] Fontaine L, Boutry C, de Frahan MH, Delplace B, Fremaux C, et al (2010) A novel pheromone quorum-sensing system controls the development of natural competence in Streptococcus thermophilus and Streptococcus salivarius. J Bacteriol 192: 1444–1454. 10.1128/JB.01251-09 20023010 PMC2820839

[9] Gardan R, Besset C, Guillot A, Gitton C, Monnet V (2009) The oligopeptide transport system is essential for the development of natural competence in Streptococcus thermophilus strain LMD-9. J Bacteriol 191: 4647–4655. 10.1128/JB.00257-09 19447907 PMC2704715

[10] Monnet V (2003) Bacterial oligopeptide-binding proteins. Cell Mol Life Sci 60: 2100–2114. 14618258 10.1007/s00018-003-3054-3 PMC11146059

[11] Desai K, Mashburn-Warren L, Federle MJ, Morrison DA (2012) Development of competence for genetic transformation of Streptococcus mutans in a chemically defined medium. J Bacteriol 194: 3774–3780. 10.1128/JB.00337-12 22609913 PMC3416567

[12] Gardan R, Besset C, Gitton C, Guillot A, Fontaine L, et al (2013) Extracellular life cycle of ComS, the competence-stimulating peptide of Streptococcus thermophilus. J Bacteriol 195: 1845–1855. 10.1128/JB.02196-12 23396911 PMC3624564

[13] de Greeff A, Wisselink HJ, de Bree FM, Schultsz C, Baums CG, et al (2011) Genetic diversity of Streptococcus suis isolates as determined by comparative genome hybridization. BMC Microbiol 11: 161 10.1186/1471-2180-11-161 21736719 PMC3142484

[14] Holden MT, Hauser H, Sanders M, Ngo TH, Cherevach I, et al (2009) Rapid evolution of virulence and drug resistance in the emerging zoonotic pathogen Streptococcus suis. PLoS One 4: e6072 10.1371/journal.pone.0006072 19603075 PMC2705793

[15] Saulnier DM, Santos F, Roos S, Mistretta TA, Spinler JK, et al (2011) Exploring metabolic pathway reconstruction and genome-wide expression profiling in Lactobacillus reuteri to define functional probiotic features. PLoS One 6: e18783 10.1371/journal.pone.0018783 21559529 PMC3084715

[16] Yang YH, Dudoit S, Luu P, Lin DM, Peng V, et al (2002) Normalization for cDNA microarray data: a robust composite method addressing single and multiple slide systematic variation. Nucleic Acids Res 30: e15 11842121 10.1093/nar/30.4.e15 PMC100354

[17] van Hijum SA, Garcia de la Nava J, Trelles O, Kok J, Kuipers OP (2003) MicroPreP: a cDNA microarray data pre-processing framework. Appl Bioinformatics 2: 241–244. 15130795

[18] Ernst J, Bar-Joseph Z (2006) STEM: a tool for the analysis of short time series gene expression data. BMC Bioinformatics 7: 191 16597342 10.1186/1471-2105-7-191 PMC1456994

[19] Saeed AI, Bhagabati NK, Braisted JC, Liang W, Sharov V, et al (2006) TM4 microarray software suite. Methods Enzymol 411: 134–193. 16939790 10.1016/S0076-6879(06)11009-5

[20] Conesa A, Gotz S, Garcia-Gomez JM, Terol J, Talon M, et al (2005) Blast2GO: a universal tool for annotation, visualization and analysis in functional genomics research. Bioinformatics 21: 3674–3676. 16081474 10.1093/bioinformatics/bti610

[21] Zdobnov EM, Apweiler R (2001) InterProScan—an integration platform for the signature-recognition methods in InterPro. Bioinformatics 17: 847–848. 11590104 10.1093/bioinformatics/17.9.847

[22] Al-Shahrour F, Minguez P, Tarraga J, Medina I, Alloza E, et al (2007) FatiGO +: a functional profiling tool for genomic data. Integration of functional annotation, regulatory motifs and interaction data with microarray experiments. Nucleic Acids Res 35: W91–96. 17478504 10.1093/nar/gkm260 PMC1933151

[23] Vickerman MM, Iobst S, Jesionowski AM, Gill SR (2007) Genome-wide transcriptional changes in Streptococcus gordonii in response to competence signaling peptide. J Bacteriol 189: 7799–7807. 17720781 10.1128/JB.01023-07 PMC2168715

[24] Peterson S, Cline RT, Tettelin H, Sharov V, Morrison DA (2000) Gene expression analysis of the Streptococcus pneumoniae competence regulons by use of DNA microarrays. J Bacteriol 182: 6192–6202. 11029442 10.1128/jb.182.21.6192-6202.2000 PMC94756

[25] Peterson SN, Sung CK, Cline R, Desai BV, Snesrud EC, et al (2004) Identification of competence pheromone responsive genes in Streptococcus pneumoniae by use of DNA microarrays. Mol Microbiol 51: 1051–1070. 14763980 10.1046/j.1365-2958.2003.03907.x

[26] Ahn SJ, Kaspar J, Kim JN, Seaton K, Burne RA (2014) Discovery of novel peptides regulating competence development in Streptococcus mutans. J Bacteriol 196: 3735–3745. 10.1128/JB.01942-14 25135217 PMC4248802

[27] Gury J, Barthelmebs L, Tran NP, Divies C, Cavin JF (2004) Cloning, deletion, and characterization of PadR, the transcriptional repressor of the phenolic acid decarboxylase-encoding padA gene of Lactobacillus plantarum. Appl Environ Microbiol 70: 2146–2153. 15066807 10.1128/AEM.70.4.2146-2153.2004 PMC383121

[28] Tran NP, Gury J, Dartois V, Nguyen TK, Seraut H, et al (2008) Phenolic acid-mediated regulation of the padC gene, encoding the phenolic acid decarboxylase of Bacillus subtilis. J Bacteriol 190: 3213–3224. 10.1128/JB.01936-07 18326577 PMC2347383

[29] Al-Shahrour F, Diaz-Uriarte R, Dopazo J (2004) FatiGO: a web tool for finding significant associations of Gene Ontology terms with groups of genes. Bioinformatics 20: 578–580. 14990455 10.1093/bioinformatics/btg455

[30] Briley K Jr, Prepiak P, Dias MJ, Hahn J, Dubnau D (2011) Maf acts downstream of ComGA to arrest cell division in competent cells of B. subtilis. Mol Microbiol 81: 23–39. 10.1111/j.1365-2958.2011.07695.x 21564336 PMC3781949

[31] Haijema BJ, Hahn J, Haynes J, Dubnau D (2001) A ComGA-dependent checkpoint limits growth during the escape from competence. Mol Microbiol 40: 52–64. 11298275 10.1046/j.1365-2958.2001.02363.x

[32] Attaiech L, Minnen A, Kjos M, Gruber S, Veening JW (2015) The ParB-parS Chromosome Segregation System Modulates Competence Development in Streptococcus pneumoniae. MBio 6. 10.1128/mBio.00662-15 PMC4488948 26126852

[33] Mirouze N, Ferret C, Yao Z, Chastanet A, Carballido-Lopez R (2015) MreB-Dependent Inhibition of Cell Elongation during the Escape from Competence in Bacillus subtilis. PLoS Genet 11: e1005299 10.1371/journal.pgen.1005299 26091431 PMC4474612

[34] Oggioni MR, Iannelli F, Ricci S, Chiavolini D, Parigi R, et al (2004) Antibacterial activity of a competence-stimulating peptide in experimental sepsis caused by Streptococcus pneumoniae. Antimicrob Agents Chemother 48: 4725–4732. 15561850 10.1128/AAC.48.12.4725-4732.2004 PMC529211

[35] Oggioni MR, Trappetti C, Kadioglu A, Cassone M, Iannelli F, et al (2006) Switch from planktonic to sessile life: a major event in pneumococcal pathogenesis. Mol Microbiol 61: 1196–1210. 16925554 10.1111/j.1365-2958.2006.05310.x PMC1618759

